# Mucolytics for Intubated Asthmatic Children: A National Survey of United Kingdom Paediatric Intensive Care Consultants

**DOI:** 10.1155/2015/396107

**Published:** 2015-02-04

**Authors:** Aarjan Peter Snoek, Joe Brierley

**Affiliations:** ^1^Department of Anaesthesia, Great Ormond Street Hospital for Children NHS Foundation Trust, Great Ormond Street, London WC1N 3JH, UK; ^2^Department of Paediatric Intensive Care, Great Ormond Street Hospital for Children NHS Foundation Trust, Great Ormond Street, London WC1N 3JH, UK

## Abstract

*Aim*. The extent to which mucolytics are utilised in mechanically ventilated asthmatic children is unknown. We sought to establish current practice in the United Kingdom (UK) including choice of mucolytic, dose, and frequency of utilisation. *Methods*. A national electronic survey was distributed to UK consultants during April and May 2014. We were able to identify 168 PICU consultants at 25 institutions to whom we were able to electronically distribute a survey, representing an estimated 81% of UK NHS PICU consultants. *Results*. Replies were received from 87 consultants at 21 institutions (response rate = 52%). Recombinant human DNase (rhDNase) does get administered by 63% of clinicians, with 54% and 19% that administer hypertonic saline or N-acetylcysteine, respectively. Of those that do administer rhDNase the majority (48%) dilute it with 0.9% saline and blindly administer it, whereas 35% administer rhDNase under bronchoscopic guidance and 17% judge the necessity for bronchoscopy according to clinical severity. 25 respondents described 7 different methods to calculate rhDNase dose. A majority (87%) of respondents expressed an interest to consider enrolling patients into an RCT that evaluates rhDNase. *Conclusion*. Significant variation exists regarding the necessity for mucolytics, choice of agent, optimal route, and dose in intubated asthmatic children.

## 1. Introduction

From Paediatric Intensive Care Audit Network (PICANet) data [1], which has its limitations such as issues surrounding coding, we believe that around 500 children are admitted with acute, severe asthma to paediatric intensive care units (PICUs) in the United Kingdom (UK) annually, of which approximately 40% are invasively mechanically ventilated. Patients with asthma develop mucus plugging and this contributes to airway obstruction with air trapping. Mucus plugging is also a significant feature on postmortems of deceased asthmatics [2] and thus attempts are sometimes made to liquefy mucus secretions of asthmatic patients in order to reduce sputum viscosity, hoping for potential reductions in morbidity, length of PICU stay, or even mortality. Such efforts include utilisation of mucolytic agents such as N-acetylcysteine (NAC), hypertonic saline (HS), or recombinant human DNase (rhDNase). However, the underlying rationale for their use exceeds the background scientific and clinical evidence, though some agents are often anecdotally hailed as panaceas. The evidence base for mucolytic drugs traverses a wide range of underlying respiratory conditions but specific studies on asthma are limited in both quantity and quality. The extent to which these agents are practically utilised on PICUs is unknown and thus we set out to establish current practice relating to the use of mucolytics for intubated asthmatic children in the UK.

## 2. Materials and Methods

The local research and development department (reference number = 14SG02) was consulted regarding a national electronic questionnaire. Approval from a research ethics committee was deemed unnecessary so the project was registered with our Clinical Audit Department (registration number = 1507) at Great Ormond Street Hospital, London. PICUs that provide level 3 National Health System (NHS) care from 26 institutions in the UK that regularly report data to the PICANet were identified. The websites of these institutions were sought for contact details of the lead clinicians or their personal assistants. This was supplemented with contact details of those personally already known to any of the authors. After having established contact with institutions via telephone or email, we were able to identify 168 PICU consultants at 25 institutions to whom we were able to distribute a survey electronically. This represents an estimated 81% of UK NHS PICU consultants (see Appendix  1 in Supplementary Material available online at http://dx.doi.org/10.1155/2015/396107). The survey was distributed in April 2014 via SurveyMonkey and was closed in May 2014 after reminder emails sent to those that had not initially responded.

## 3. Results

Replies were received from 21 institutions with a survey response rate of 52% (*n* = 87). Having surveyed an estimated 81% of UK NHS PICU consultants, we thus received replies from an estimated 42% of all UK NHS PICU consultants. Of the 87 PICU consultant responses, 15% held a position of head of department or clinical lead. A summary of the survey questions is displayed in Table 1 and a list of institutions that responded is displayed in Appendix  2.

### 3.1. Administration of rhDNase

RhDNase is never administered to children intubated with asthma by 37% (*n* = 87) of respondents. Amongst the 63% that do administer rhDNase, the majority (64%) do so only occasionally (to less than one-third of intubated asthmatics).

### 3.2. Administration of Hypertonic Saline (HS)

HS is never administered to children intubated with asthma by 46% (*n* = 85) of respondents. Amongst the 54% that do administer HS, the majority (72%) administer HS only occasionally.

### 3.3. Administration of N-Acetylcysteine (NAC)

A high proportion (81%) of consultants never administer NAC to intubated asthmatics. The majority (88%) of those that do administer NAC do so only occasionally.

### 3.4. Route of rhDNase Administration

Instillation of rhDNase down the endotracheal tube (ETT) of an intubated asthmatic was the preferred route of administration for 42% (*n* = 55). Administering rhDNase as a nebulised solution via the ETT was designated by 56%, though this was qualified by 2 respondents in the fact that if persistent collapse or focal change was present then rhDNase would rather be introduced via ETT. Two percent did not commit to a specific route.

### 3.5. Endotracheal Installation Method

Of those that do administer endotracheal rhDNase (*n* = 23), 48% dilute the mucolytic with 0.9% saline and blindly administer it, whereas 35% administer rhDNase under bronchoscopic guidance. The remaining 17% commented that the necessity for bronchoscopy would be dictated by the patient's clinical severity.

### 3.6. Dose of Endotracheal rhDNase

Concerning the dose of endotracheal rhDNase, 25 respondents described a total of 7 different methods to calculate the dose of rhDNase (Table 2). Factors that were considered in these computations included the patient's age, weight, or body surface area (BSA).

### 3.7. Chest Physical Therapy

Chest physiotherapy would be requested to occur on at least one-third of intubated asthmatic children by 84% (*n* = 85) of respondents and 37% would request chest physiotherapy for all intubated patients. In contrast, only 1% would never request the involvement of a chest physiotherapist.

### 3.8. Randomised Controlled Trial (RCT) Interest

A willingness to consider enrolling intubated asthmatic patients into an RCT that evaluates rhDNase was conveyed by 87% (*n* = 85). The substance to which rhDNase should be compared in an RCT was reckoned to be 0.9% saline for 69% of respondents, HS for 15%, and NAC for 4%. The remaining 12% of respondents described other opinions such as comparing standard care or no endotracheal substance or performing a study with 3 or more arms.

## 4. Discussion

Our inability to survey approximately one-fifth of UK PICU consultants limits our findings but we achieved a satisfactory survey response rate (52%) and believe that our results, although imperfect, represent a reasonable description of current practice.

### 4.1. Administration of rhDNase

Sputum in asthma has an increased DNA content, so there is biological plausibility that rhDNase may also benefit asthmatics by digesting extracellular DNA. In lower quality studies rhDNase has been successfully used in both intubated [3–7] and unintubated [8, 9] asthmatic children. In intubated adults some studies have shown favourable outcomes with rhDNase [10, 11] but other studies have not been able to reproduce these successes in both adults [12] and children [13, 14]. Our survey findings of equipoise for rhDNAse, with just over 60% that administer rhDNase to intubated asthmatics but nearly 40% that do not, are congruous with the equivocal evidence on rhDNase efficacy as well as the lack of high-quality evidence.

### 4.2. Administration of Hypertonic Saline (HS)

HS is utilised diagnostically to elicit airway hyperresponsiveness and can cause airway obstruction possibly via neurogenic reflexes [15]. Nonetheless, due to its mucolytic properties HS has been explored as a therapeutic possibility for mucus hypersecretory diseases such as cystic fibrosis (CF) [16]. HS for bronchiolitis has been fairly extensively studied and favourable effects have been shown [17, 18] though due to conflicting results from other trials [19] the role of HS remains unclear even in infants with bronchiolitis [20]. HS can enhance mucociliary clearance [21] but with concerns of eliciting airway hyperresponsiveness and with minimal evidence for clinical improvement it is perhaps unsurprising that we found equipoise to exist regarding administration of HS to intubated asthmatics, with 46% that never administer it.

### 4.3. Administration of N-Acetylcysteine (NAC)

Antioxidant properties of NAC are thought to be useful in targeting the overwhelming oxidative stress [22] associated with asthma. Additionally NAC possesses mucolytic properties in its ability to break disulphide bonds with a potential to break down mucus into smaller, less viscous units. However, patients can rarely develop worsening asthma [23] and evidence for its benefit in both CF [24] and asthma is lacking in both quality and quantity. The reason why we found that just over 80% of consultants never administer NAC to intubated asthmatics is likely to be related to concerns for bronchospasm as well as insufficient evidence for a benefit with NAC.

### 4.4. Route and Dose of rhDNase Administration

Multiple authors have previously described intratracheal rhDNase administration [3–7, 25–27], often blindly via a feeding catheter. Nebulised rhDNase is a familiar route of administration for CF patients and this therapeutic modality has been tried in asthmatic patients too [10, 11] though little evidence exists in children and has not always shown benefit [13]. Despite the paucity of evidence for rhDNAse in asthmatic children, it is conceivable that familiarity with this route of administration for other conditions [28] reflects that 56% of survey respondents would administer rhDNase as a nebulised solution rather than via intratracheal instillation as a solution. Anecdotal reports exist for bronchoscope-guided rhDNase administration to facilitate cast removal [29] or to target specific collapsed lung areas. However, bronchoscopy may not necessarily confer an advantage over standard therapy alone [30] and in our opinion a technique of blind administration has the appeal of being more feasible. This practical aspect has probably contributed to shaping our findings that, of those respondents who use rhDNase, approximately only 1 in 3 (35%) always administer it under bronchoscopic guidance.

Use of off-label or unlicensed drugs is not uncommon in paediatric practice and rhDNase falls within this remit with challenging consequences for establishing optimal dose. A diversity of intratracheal doses have been utilised in previously published studies, with 4 mg·m^−2^ body surface area (BSA) commonly used [29]. Surprisingly no survey respondents indicated the use of this dose yet seven different methods to calculate rhDNase dose were described, with no one method particularly overrepresented. The large variation in current practice reflects the lack of data on this matter with ongoing unknowns.

## 5. Conclusions

There are marked variations in practice amongst UK PICU consultants with regard to the administration of mucolytics in critically ill asthmatic children. The underlying reasons are diverse although a lack of good quality evidence seems to be a significant factor, with clear equipoise about the benefits of HS and rhDNase for intubated asthmatic children.

There is a lack of consensus about either the optimal route or the dose of rhDNase when used for this indication. We believe this survey demonstrates that clinicians are unsure about the use of mucolytics in paediatric patients requiring mechanical ventilation for acute, severe asthma. The widespread yet disparate utilisation of these agents calls for at the very least a formal prospective audit of practices. Their optimal use, not least which treatment confers the greatest clinical benefit, should be determined via either a prospective clinical study or comparative-effectiveness trial. Many unanswered questions remain regarding asthma management such as ventilation strategies or the place for adjunctive therapies such as chest physiotherapy or mucolytics. We have shown that significant appetite exists in the UK for an RCT to compare rhDNase with placebo and current equipoise on the role of mucolytic agents implies that such a trial would be useful to clinicians.

## Supplementary Material

A database of contact details for all PICU consultants in the UK unfortunately does not exist, which presented a challenge regarding survey distribution and response rate calculations that impact on survey validity. We believe that we managed to distribute an electronic survey to 81% of UK NHS PICU consultants and the calculation for this can be seen in Appendix 1.
We are grateful to the respondents from 21 institutions in the UK that provide level 3 paediatric intensive care services. The institutions from which at least one survey response was received are listed in Appendix 2.

## Figures and Tables

**Table 1:** Summary of survey questions and responses.

What is your title? (*n* = 88)	Head of department or clinical lead	15%
	Other PICU consultants	85%

An asthmatic child has been intubated and admitted to your PICU with acute, severe asthma: would you prescribe rhDNase? (*n* = 87)	Never	37%
	Occasionally	40%
	Sometimes	16%
	Usually/often	6%
	Always	1%

What route of rhDNase administration would you use? (*n* = 54)	Nebulisation (via ETT)	56%
	*Intratracheal *	
	(i) Blind, diluted with saline	20%
	(ii) Bronchoscopic guidance	15%
	(iii) Depends on clinical condition	7%
	No opinion	2%

If this child were to receive intratracheal rhDNase, what would be the optimal dose? (*n* = 25)	No opinion	44%
	2 mg/m^2^ BSA	4%
	4 mg/m^2^ BSA	0%
	0.1 mg/kg	0%
	0.2 mg/kg	16%
	Other	36%

Would you prescribe/administer intratracheal hypertonic saline? (*n* = 85)	Never	46%
	Occasionally	39%
	Sometimes	13%
	Usually/often	2%
	Always	0%

Would you prescribe/administer intratracheal NAC (N-acetylcysteine)? (*n* = 85)	Never	81%
	Occasionally	17%
	Sometimes	1%
	Usually/often	1%
	Always	0%

Would you request a chest physiotherapist to treat the patient? (*n* = 85)	Never	1%
	Occasionally	15%
	Sometimes	18%
	Usually/often	29%
	Always	37%

Would you be willing to consider enrolling intubated asthmatic patients admitted to your PICU into a rhDNase trial? (*n* = 85)	Yes	87%
	No	13%

If an RCT was undertaken in intubated asthmatic children, with one group receiving intratracheal instillation of rhDNase, what intratracheal substance should the control group receive? (*n* = 85)	Placebo (0.9% NaCl)	69%
	NAC	4%
	Hypertonic saline	15%
	Other	12%

Key: “Never” is 0%; “occasionally” is <33% of cases; “sometimes” is 33%–66% of cases; usually/often is >66% of cases; always is 100% of cases.

**Table 2:** Variety of methods in current practice to calculate dose of intratracheal recombinant human DNase.

Basis for calculation	Dose
Variable dose based on body surface area	2 mg/m^2^

Variable dose based on weight	0.2 mg/kg
	0.25 mg/kg
	0.25 mg/kg but only up to a maximum of 5 mg
	0.1 mg/kg if >10 kg or 0.25 mg if <10 kg (diluted)

Fixed dose but diluted to a volume based on weight	2.5 mg diluted in saline to volume of 1 mL/kg
	2.5 mg diluted to either 10 mL or 50 mL (depends on size)
