# Supplementary material for: Mucolytics for Intubated Asthmatic Children: A National Survey of United Kingdom Paediatric Intensive Care Consultants
Source: Crit Care Res Pract. 2015 Feb 4;2015:396107. doi: 10.1155/2015/396107 (PMC4334628; doi:10.1155/2015/396107)
Supplement: Supplementary file 1 — A database of contact details for all PICU consultants in the UK unfortunately does not exist, which presented a challenge regarding survey distribution and response rate calculations that impact on survey validity. We believe that we managed to distribute an electronic survey to 81% of UK NHS PICU consultants and the calculation for this can be seen in Appendix 1. We are grateful to the respondents from 21 institutions in the UK that provide level 3 paediatric intensive care services. The institutions from which at least one survey response was received are listed in Appendix 2. [file 396107.f1.pdf]

Appendix 1

Number of consultants that work in National Health System (NHS) paediatric intensive care units that provide level 3 care

We believe that through a contact person at each institution we were able to identify the email addresses of all PICU consultants at 21 institutions. 168 surveys were sent out to all PICU consultants working at these 21 institutions.

We utilised data from PICANet (2) to estimate that 5 consultants work at 1 institution where we were unable to gain even just one PICU consultant's email address. At 4 institutions we were able to obtain the email address of 1 consultant at each institution and a survey was sent to this contact. We estimate that a total of 34(5 + 13 + 8 + 8) other PICU consultants work at these 4 institutions. We were unable to obtain their contact details and thus 34 were never sent a survey.

The summation of 168, 5, and 34 gives an estimated denominator of 207. Consequently we estimate that 81% of consultants that work in NHS PICUs that provide level 3care were sent a survey.

Appendix 2

Institutions from which survey responses were received

Alder Hey Children’s NHS Foundation Trust

Barts Health NHS Trust

Belfast Health and Social Care Trust

Birmingham Children’s Hospital NHS Foundation Trust

Cardiff &Vale University Health Board

Central Manchester University Hospitals NHS Foundation Trust

Great Ormond Street Hospital for Children NHS Foundation Trust

Guy’s and St Thomas’ NHS Foundation Trust

Imperial College Healthcare NHS Trust

King's College Hospital NHS Foundation Trust

Leeds Teaching Hospitals NHS Trust

The Newcastle upon Tyne Hospitals NHS Foundation Trust

-Freeman Hospital

-Great North Children’s Hospital

NHS Greater Glasgow and Clyde

NHS Lothian

Nottingham University Hospitals NHS Trust

Oxford University Hospitals NHS Trust

Sheffield Children’s NHS Foundation Trust

St George's Healthcare NHS Trust

University Hospital of North Staffordshire NHS Trust

University Hospital Southampton NHS Foundation Trust
